# Portable pressure chamber for manual camera-assisted monitoring of leaf water potential

**DOI:** 10.1016/j.ohx.2025.e00632

**Published:** 2025-02-25

**Authors:** Willian Viana Campos, José Teixeira Filho, Alcebiades Rebouças São José

**Affiliations:** a Faculty of Agricultural Engineering, State University of Campinas, Campinas, São Paulo, Brazil; b Department of Phytotechnics, State University of Southwest Bahia, Vitória da Conquista, Bahia, Brazil

**Keywords:** Pressure chamber, Water potential, Manual monitoring

## Abstract

This paper presents a detailed description of the assembly and components of a pressure chamber used to measure leaf water potential in leaves, particularly in african mahogany. The assembly and components of this pressure chamber were specifically designed for field experiments. The image capture camera plays a crucial role in recording changes occurring in the sample during measurements. It can also be coupled with image analysis devices to quantify alterations in leaf cells and stomata under varying pressure levels. A cylindrical acrylic support ensures the stability of the camera during the procedure, providing additional protection. The pressure chamber assembly is designed to securely accommodate leaves during measurements, facilitating the manipulation of gas levels and enabling the creation of a controlled atmosphere within the chamber. The sealing valve is an essential component, allowing precise control of internal pressure, preventing leaks, and enabling the study of plant responses under different atmospheric conditions. This pressure chamber proven to be a valuable tool in plant ecophysiology, enabling precise monitoring of plant responses to diverse environmental conditions. Consequently, it contributes significantly to the conservation and sustainable management of african mahogany and other plant species in their natural environments.

## Graphical abstract

**Figure:**
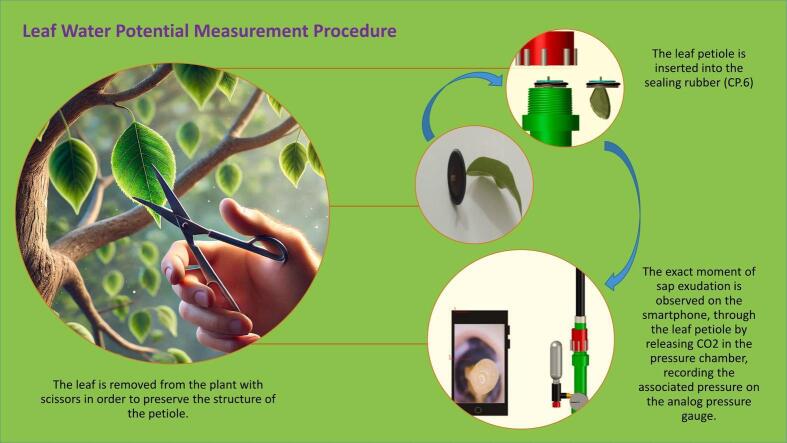

Specifications Table.

**Table:** 

Hardware name	PORTABLE PRESSURE CHAMBER FOR MANUAL CAMERA-ASSISTED MONITORING OF LEAF WATER POTENTIAL
Subject area	Sciences environmental, planetary and agricultural
Hardware type	Measurements field and sensors
Closest commercial analog	SCHOLANDER CHAMBER
Open source license	CC BY 4.0
Cost of hardware (CAD$)	Cost approximated to US$ 20.00.
Source file repository	Not applicable
Operating Instructions	https://youtu.be/xsTd-J843Ps?si=HaLn3oTeaSLPfkzW

## 1 Hardware in context

Leaf water potential is an essential parameter in plant ecophysiology, providing insight into the water status of plant leaves and their interactions with environmental conditions. It is of great significance because it directly indicates the hydration status of the plant and its responses to environmental conditions, including water stress [1]. Leaf water potential serves as a sensitive indicator of plant water status, representing the sum of all forces influencing water movement within a leaf [2]. This parameter measures the energy potential of water in relation to its free movement within a system, expressed in negative values, as water flows from higher (less negative) to lower (more negative) water potential [3].

Determining leaf water potential is crucial for understanding plant behavior under water stress and for optimizing irrigation management in agricultural systems [4]. Accurate measurements allow farmers to assess plant hydration and make informed decisions about irrigation timing and quantity, minimizing water waste while preventing stress that could harm crop development and productivity [5].

Beyond agriculture, leaf water potential is a critical parameter in plant ecology, providing insights into plant responses to various environmental conditions such as climate change, seasonal variations, and fluctuating water availability [6]. It also facilitates studies on plant behavior concerning water supply and stress, with implications for terrestrial ecosystem functioning [7]. Accurate measurement of leaf water potential is therefore essential for irrigation management and the study of plant ecophysiology under diverse environmental scenarios [8]. Continued research in this area is fundamental for water resource conservation and understanding the impacts of environmental changes on ecosystem dynamics.

A pressure chamber, also known as a Scholander chamber, is a widely used device for measuring leaf water potential [9]. However, traditional pressure chambers are expensive and not very accurate, as they rely on analog visualization of sap exudation through a magnifying glass.

The principle of the pressure chamber is based on the relationship between the water pressure within leaf cells and external atmospheric pressure. When a leaf is well-hydrated, the water pressure in its cells is high, resulting in a less negative water potential (closer to zero). Conversely, under water stress, the pressure in leaf cells drops, leading to a more negative water potential [10].

To improve measurement accuracy and practicality, the equipment described in this study digitally measures leaf water potential, capturing the exact moment of sap exudation through a digital image capture camera integrated with a mini pressure chamber pressurized by a 16-gram CO_2_ capsule. This innovative equipment enables detailed study of plant responses to water stress, offering valuable data for water resource conservation.

The proposed camera-assisted manual monitoring pressure chamber is composed of key components such as a pressure chamber, a CO_2_ capsule, and a micro-image capture camera. Its operation involves applying pressure to a plant sample inside the chamber and recording the pressure required to release water from the cut end, which reflects the plant's water status. This design utilizes digital technology (micro-camera) to ensure accurate and reliable measurements of plant water dynamics, focusing on its technical functionality and operational efficiency.

## 2 Hardware description

The Portable Pressure Chamber for Camera-assisted Manual Monitoring of Leaf Water Potential was developed to address limitations in existing pressure chamber technologies by offering improved precision, portability, and cost-effectiveness. Unlike traditional devices, this system utilizes a tubular digital micro-camera to capture high-resolution images of the leaf petiole in real-time, enabling the precise observation of the sap exudation process. The device comprises a mini pressure chamber for leaf insertion, a CO_2_ capsule for controlled air release, a gas-sealing valve, and an analog pressure gauge to monitor system pressure. The integration of the digital micro-camera with the pressure chamber eliminates the subjectivity of analog observations, providing continuous and accurate monitoring of plant water dynamics. These features collectively represent a significant advancement over conventional pressure chambers.

The mini pressure chamber is compact and portable, constructed from lightweight yet durable materials to facilitate transportation and field use. Despite its slim and portable design, the chamber maintains full functionality, enabling accurate measurements in diverse environments. CO_2_ is used as a compression gas in the system and the CO_2_ capsule is coupled with a precision valve that allows gradual and controlled gas release into the pressure chamber, ensuring consistent and reliable measurements.

A key innovation of this equipment is the integration of a digital image capture system with an tubular digital micro-camera. This system records the exact moment of sap exudation and is linked to analysis software that processes and stores data for subsequent interpretation. This configuration enables precise monitoring by eliminating the subjectivity associated with traditional analog visual observation. The equipment includes a lightweight design, a CO_2_ capsule for energy efficiency, and is portable, making it suitable for fieldwork.

This equipment it is designed to be cost-effective and adaptable for use in a range of research projects, measuring water potential across diverse plant species. The proposed camera-assisted manual monitoring system for leaf water potential operates through the integration of its main components: the tubular digital micro-camera, the digital image capture system, and the analysis software. The system processes data in real-time, offering researchers a reliable tool for assessing plant water potential under various conditions.

## 3 Design files Summary

**Table:** 

**File name of project**	**File type**	**License of code open**	**Location from the file**
File of project 1	CAD files	CC BY 4.0	Viana Campos, Willian (2024), “DESIGN FILES − PORTABLE PRESSURE CHAMBER FOR DIGITAL MONITORING OF LEAF WATER POTENTIAL”, Mendeley Data, V1, https://doi.org/10.17632/53fxdzggpk.1

File of project 1: Detailed three-dimensional design of the pressure chamber and image receiver device (smartphone) with the assembled components. The file contains detailed representations of the parts and system assembly, including the pressure chamber and the image receiver device, with all components and their interactions in the layout.

## 4 Bill of materials Summary

**Table:** 

**Designator**	**Component**	**Number**	**Cost per unit − coin**	**Material of type**	**Source of materials**
**CP.1**	Image capture camera (tubular digital micro-camera)	1	US$ 5	−Metal-Semiconductor-Polymer	
**CP.2**	Capture Camera Support	1	US$ 0.2	−Polymer	
**CP.3**	12.5 mm diameter base tube of capture camera mount	1	US$ 0.2	−Polymer	
**CP.4**	Pressure valve with 12.5 mm glove base and internal thread	1	US$ 0.2	−Polymer	https://www.hidrauconex.com/luva-pvc-solda-rosca-lr-de-20 mm-x-12
**CP.5**	16 g CO_2_ cylinder	1	US$ 1.5	−Metal	
**CP.6**	Sealing rubber	1		−Polymer	
**CP.7**	Analog Pressure Gauge (1 MPa)	1	US$ 5	−Polymer-Metal	https://produto.mercadolivre.com.br/MLB-735867314-manmetro-horizontal-rosca-18-bsp-escala-0-a-10-bar-150-psi-_JM
**CP.8**	Pressure Chamber Tube (12.5 mm diameter −15 cmlong)	1	US$ 0.2	−Polymer	
**CP.9**	CO_2_ regulation valve	1	US$ 5	−Polymer-Metal	https://www.mercadolivre.com.br/bomba-inflador-pneu-co2-16 g-presta-schrader-bike-mtb-speed-cor-preto/p/MLB26090016
**CP.10**	12.5 mm metal washer	1	US$ 0.1	−Metal	
**CP.11**	12.5 mm O-ring	1	US$ 0.2	−Rubber	
**CP.12**	12.5 mm Silicone Rubber Ring	1	US$ 1,7	−Silicone	https://doutorirrigacao.com.br/medicao-e-instrumentacao/rolha-*para*-tensiometro-do-doutor-irrigacao--p?
**CP.13**	12.5 mm Polymer Washer	1	US$ 0.2	−Metal	
**CP.14**	12.5 mm Metal Washer	1	US$ 0.2	−Metal	
**CP.15**	Glove for 12.5 mm capture camera mount with external thread	1	US$ 0.2	−PVC	
**CP.16**	12.5 mm PVC pipe cap	1	US$ 0.2	−PVC	
**MM.1**	Super glue	1	US$ 1.5	−Polymer	

Designators: CP (components of equipment). MM (material used to assemble the equipment)

## 5 Build Instructions

The first step in the assembly involves coupling the CO_2_ regulation valve (CP.9) to the pressure chamber tube (CP.8). This requires a CO_2_ inspect valve and a 15 cm 12.5 mm diameter PVC tube (CP.8). The CO_2_ regulation valve is connected to the PVC tube by screwing the valve tip into the tube after creating a hole matching the valve tip's diameter. It is essential to ensure all connections are tightly sealed to prevent air leaks.

**Fig. 1:**
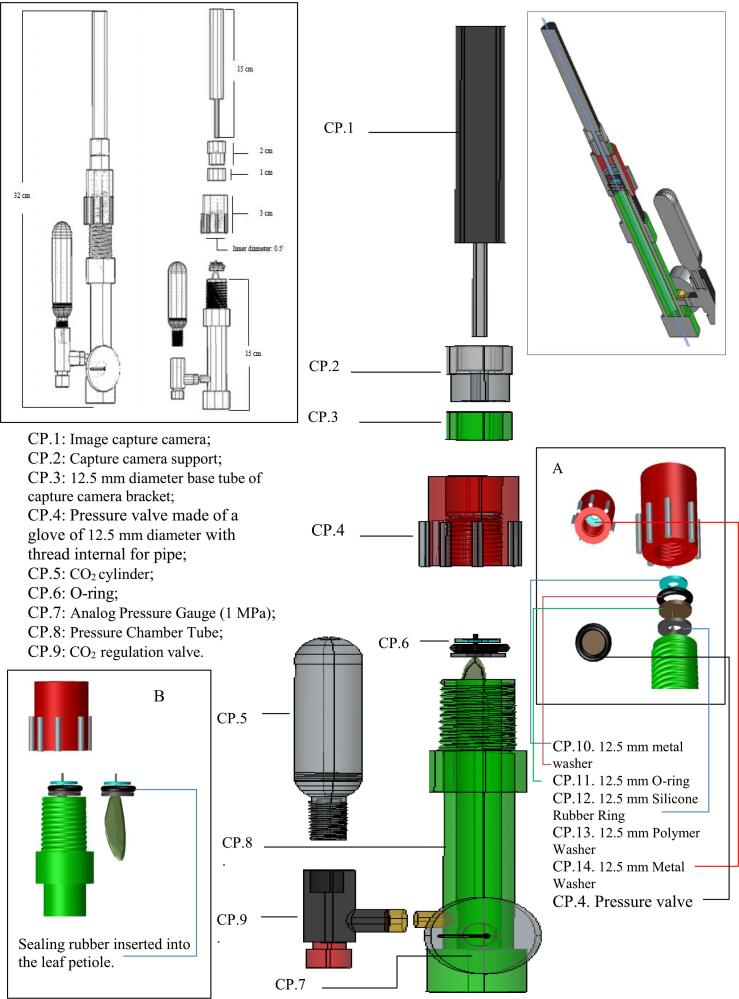
Pressure chamber with its components highlighted separately.

In the second step, the pressure chamber tube (CP.8) is connected to a 12.5 mm diameter sleeve with external pipe threads (CP.15). A threaded terminal is necessary on the pressure chamber tube to allow the threading of the pressure valve (CP.4). To achieve this, the high-resistance tube (CP.8) is connected to the 12.5 mm diameter sleeve using instant glue (Designator MM.1), as illustrated in Fig. 1. This tubular base forms the primary structure where the pressure valve will be attached. The system generates pressure by compressing the threaded terminal part of the pressure chamber tube against the internal support of the sealing valve. This arrangement expands the sealing rubber under the leaf petiole stem, ensuring that air is expelled only through the leaf petiole's microvessels. This enables sap capture using the tubular digital micro-camera.

The micro-camera used is a Smart Visual Ear Pick (Fig. 1. – CP.1), featuring the following specifications: resolution = 1080p or higher; magnification range = 100x; connectivity = wireless, allowing data transfer to portable devices; power source = battery-operated for enhanced mobility; weight = 13 g; size = 145 mm in length. This micro-camera was selected for its portability, cost-effectiveness, and ability to capture high-resolution images suitable for monitoring sap exudation in the field. Its compact design and lightweight nature make it ideal for integration into a mobile setup.

**Fig. 2:**
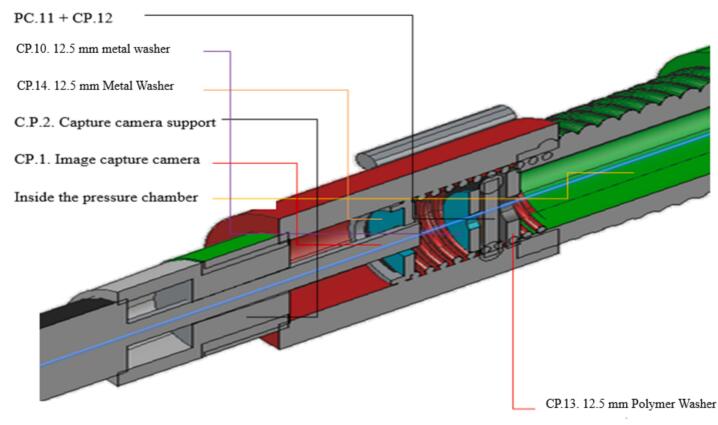
Drawing with a three-dimensional cross-sectional view, including an enlargement of the coupling between the pressure valve (CP.4) and the image capture camera (CP.1), showing its internal components.

The third step involves assembling the leaf into the rubber sealing system. The rubber sealing components (CP.10, CP.11, CP.12, and CP.13) are joined in the sequence shown in Fig. 1 (A). The plant leaf is inserted between the rubber sealing components, ensuring it is centered within the rubber sealing to allow the sap to be observed clearly by the image capture camera (CP.1). The setup must facilitate easy replacement and adjustment of the leaf. See (Fig. 2).

In the fourth step, the sealing rubber with the attached leaf is coupled to the pressure chamber tube (CP.8). The leaf and sealing rubber are placed at the end of the pressure chamber tube, as shown in Fig. 1 (B). Proper sealing is critical to maintain system pressure and prevent leaks.

The fifth step connects the pressure valve (CP.4) to the threaded end of the pressure chamber tube (CP.8). The valve is threaded onto the end of the tube, ensuring that the plant leaf is already positioned within the rubber seal and the pressure chamber tube from the previous step. The pressure valve regulates the controlled entry and exit of air, maintaining accurate pressure within the system.

The sixth step integrates the digital image capture camera (CP.1) into the system. The 12.5 mm diameter base tube of the capture camera support (CP.3) and the capture camera support (CP.2) are attached to the anchoring tubular base beneath the pressure valve. The camera is positioned to directly observe the leaf petiole, and its focus is adjusted to capture the sap exudation point. Finally, the seventh step connects the analog pressure gauge (CP.7 − Technical specifications: Horizontal pressure gauge, 3,12 mm thread, 0 to 1 MPa scale, 150 Psi maximum pressure) to the pressure chamber tube (CP.8).

The pressure gauge is attached by screwing its outlet tip into the 15 cm 12.5 mm diameter high-resistance PVC tube after creating a hole matching the gauge tip's diameter. All connections must be carefully sealed to ensure no air leaks occur.

### 5.1 Tests and calibration

The testing and calibration process begins with the initial pressurization. The gas release valve of the CO_2_ capsule and the pressure chamber are inspected to ensure proper pressurization. Adjustments are made as needed to achieve the desired pressure, and it is essential to verify that there are no leaks in the system.

The next step involves calibrating the image capture system. The micro-camera is adjusted to ensure it correctly focuses on the sap exudation point. Additionally, the image capture system is tested to confirm it accurately records the moment of sap exudation.

### 5.2 Safety measures

Safety measures are essential during the assembly and operation of the device. All pressure connections must be thoroughly inspected to prevent leaks that could lead to accidents. Additionally, appropriate protective equipment, such as safety glasses and gloves, should always be used to ensure safe handling of the device.

## 6 Operating Instructions

### 6.1 Step 1: Equipment preperation

Step 1 involves preparing the equipment to ensure proper functionality and accurate data collection. Begin with a thorough inspection of all equipment components, including the pressure chamber, the CO_2_ regulation valve and CO_2_ cylinder assembly, and the image capture system. Confirm that all connections are secure and inspect for any signs of wear or damage.

Next, proceed to couple the leaf into the pressure chamber. Start by placing the leaf petiole into the sealing rubber, ensuring it is positioned correctly for clear observation by the digital image capture camera. Once the leaf is properly inserted into the sealing rubber through its petiole, place the sealing rubber with the leaf into the Pressure Chamber Tube (CP.8). Screw the Sealing Valve (CP.4) into the Pressure Chamber Tube in such a way that it applies sufficient pressure to the sealing rubber, allowing the chamber to be pressurized. Ensure that the air escapes only through the vessels in the leaf petiole.

Finally, connect the digital image capture camera (CP.1) to the sealing valve (CP.4) to monitor sap exudation. Turn on the digital camera by pressing its power button, and establish a connection to a cell phone via the HND app, using a Wi-Fi signal. Adjust the camera to focus on the leaf, ensuring the lens is clean and unobstructed. Use the focus controls in the app to achieve a clear view of the sap exudation point.

### 6.2 Step 2: Operation equipment

Step 2 involves the operation of the equipment to monitor and capture data effectively. Begin by connecting the digital image capture camera to the smartphone via Wi-Fi. Once connected, open the HND image capture software and adjust the settings as needed to achieve the best image quality. Gradually open the CO_2_ release valve to fit the CO_2_ pressure to the Pressure Chamber Tube (CP.8), ensuring a steady and controlled release of gas.

Monitor the pressure using the analog pressure gauge to keep it within the desired range. Through the digital camera (CP.1), observe the leaf and focus on the point of sap exudation. Use the HND image capture software to document the exact moment of exudation. At this critical point, close the CO_2_ release valve to stop the airflow into the Pressure Chamber Tube (CP.8), and record the pressure displayed on the analog pressure gauge. This pressure corresponds to the exact moment of sap exudation in the leaf petiole, as observed in the images displayed on the smartphone.

**Fig. 3:**
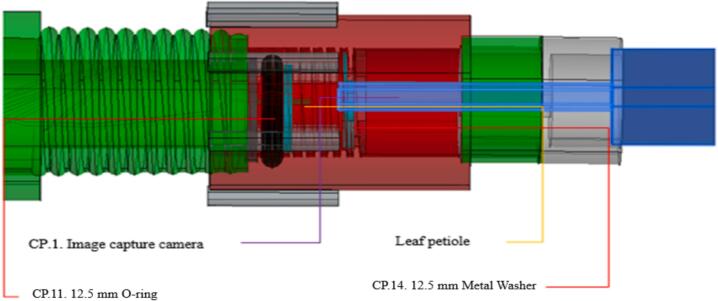
Three-dimensional side drawing of the coupling system between the Pressure Chamber Tube (CP.8) and the Pressure Valve (CP.4), to which the image capture camera is attached.

The moment of sap exudation through the leaf petiole occurs when the sap begins to flow, identified by the wetting of the petiole surface under the pressure exerted by the CO_2_ in the pressure chamber (CP.8). This pressure is recorded on the analog manometer. The exact point at which the petiole becomes wet should be used to record the manometer reading, representing the leaf's water potential. After this initial wetting, sap bubbling through the petiole begins, indicating that the pressure exceeds the characteristic point correlated to the leaf's water potential. See (Fig. 3).

Finally, save the captured images and data within the software, ensuring that all information is stored correctly for later analysis. This process ensures accurate and reliable data collection for subsequent evaluations.

### 6.3 Step 3: Operation finalization

Begin by carefully unscrewing the pressure valve (CP.4) from the Pressure Chamber Tube (CP.8), allowing the air within the system to be safely released. Once the system is depressurized, remove the leaf along with the sealing rubber from the Pressure Chamber Tube (CP.8). Finally, separate the leaf from the sealing rubber (CP.6), completing the disassembly process and ensuring the equipment is ready for subsequent use or maintenance.

### 6.4 Potential security risks

The device was tested up to its breaking point, with a maximum recorded pressure of 250 PSI. The CO_2_ cylinder is sold already pressurized, maintaining the gas in a liquid state due to its internal pressure. However, the maximum safe pressure for the pressure chamber tube (CP.8) is limited to 150 PSI. The pressure release valve is manually and directly operated on the equipment, and no leaks were detected during its use. High pressure poses a significant risk, as leaks or failures in the pressure chamber can lead to explosions or accidents. To mitigate this, it is essential to inspect all connections and seals prior to operation, avoid exceeding the recommended pressure, and continuously monitor the system, ensuring the maximum pressure does not exceed 0.9 MPa. Improper handling of the leaf can cause damage to its leaf structure and petiole, which would compromise the integrity required to obtain the water potential. To avoid such damage, handle the leaf with care and attention. Lastly, the image capture equipment is vulnerable to damage from impacts or moisture, potentially causing data loss. To safeguard this system, it should be protected from environmental hazards and undergo regular maintenance inspects to confirm proper functionality.

## 7 Validation and characterization

### 7.1 Case of use relevant: Study of water potential leaf in mahogany african (*Khaya senegalensis*)

The camera-assisted manual monitoring system is designed to measure the leaf water potential of plants, specifically african mahogany leaves (*Khaya senegalensis*), in a research project focused on the ecophysiological behavior of young *Khaya senegalensis* plants in a saline environment. This study is critical for understanding how these plants respond to water stress, which is essential for water conservation and developing climate change adaptation strategies.

The goal of the study was to determine both the direct and indirect effects of environmental interactions on the leaf transpiration, stomatal conductance, and photosynthesis of Khaya senegalensis (These physiological parameters were obtained using an infrared gas analyzer − IRGA) in a semiarid region. The research was conducted using drainage lysimeters with young african mahogany plants (*Khaya senegalensis*), with three experimental units (plants), irrigated with water at an electrical conductivity of 0.5 dS/m to optimize water use in a semiarid environment. Key environmental variables influencing the ecophysiological behavior of the mahogany plants included global radiation (Rg), photosynthetically active radiation (Qleaf), atmospheric vapor pressure deficit (VPD), and leaf water potential. The experiment was conducted during the first four months of plant development, with data collection across three consecutive months: january, february, and march 2022. These data were collected from 7:00 a.m. to 6:00p.m., with a one-minute interval for data collection on each plant.

### 7.2 Collect and analysis of data − water potential leaf (Ψw)

During the experimental period, three leaves from each plant were collected from the middle third of the aerial part to evaluate the leaf water potential using the Pressure Chamber equipment. To remove the plant leaf, any cutting tool, such as a blade or scissors, can be used, ensuring that the cross-section of the petiole (cut section of the petiole) is smooth. However, if the cut is not perfectly made, it will not compromise the measurement of the water potential. The leaves were collected early in the morning, and the leaf water potential was determined following the method outlined by [11]. Fig. 5 illustrates the relationship between soil water potentials and plant leaf transpiration during different periods of water restriction. Water potentials ranged from −0.2 MPa to −0.5 MPa, reflecting various levels of water availability, with leaf water potential values, recorded by the equipment's analog pressure gauge in kgf/cm^2^, multiplied by the factor 0.0981 to be transformed into MPa values. The more negative the water potential, the greater the water deficit available to plants, which limits the absorption and transport of water from the soil to the shoots.

**Fig. 4:**
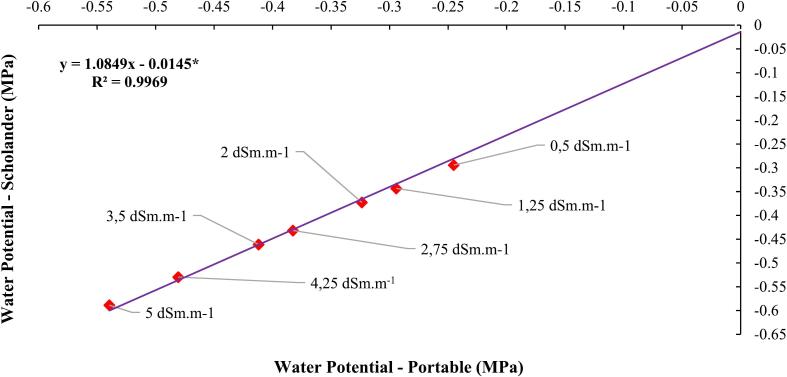
Linear correlation between the water potentials measured with the portable manual monitoring equipment assisted by a camera and the Scholander chamber for increasing levels of electrical conductivity of the irrigation water. * Significant by Tukey's test at a 5 % probability level (p < 0.05).

The leaf water potential measurements were taken simultaneously using two measurement methods. The same leaf was subjected to water potential measurement using the Portable Pressure Chamber and then the Scholander Pressure Chamber, with water potential values being higher by a factor of 0.05 MPa for data obtained through the Scholander Pressure Chamber (Fig. 4). This difference in values arises because the point of observation of the exudation of sap from the leaf petiole is observed at a lower pressure with the Portable Pressure Chamber compared to the Scholander Pressure Chamber, due to the precision in observation. When sap begins to be exuded from the petiole, the Portable Pressure Chamber can already detect the exudation (which occurs at a lower pressure), whereas the exudation observation using the Scholander Pressure Chamber is only possible under higher pressures, when sap bubbling is observed under the petiole, and this is viewed through a magnifying lens, which reduces its accuracy.

**Fig. 5:**
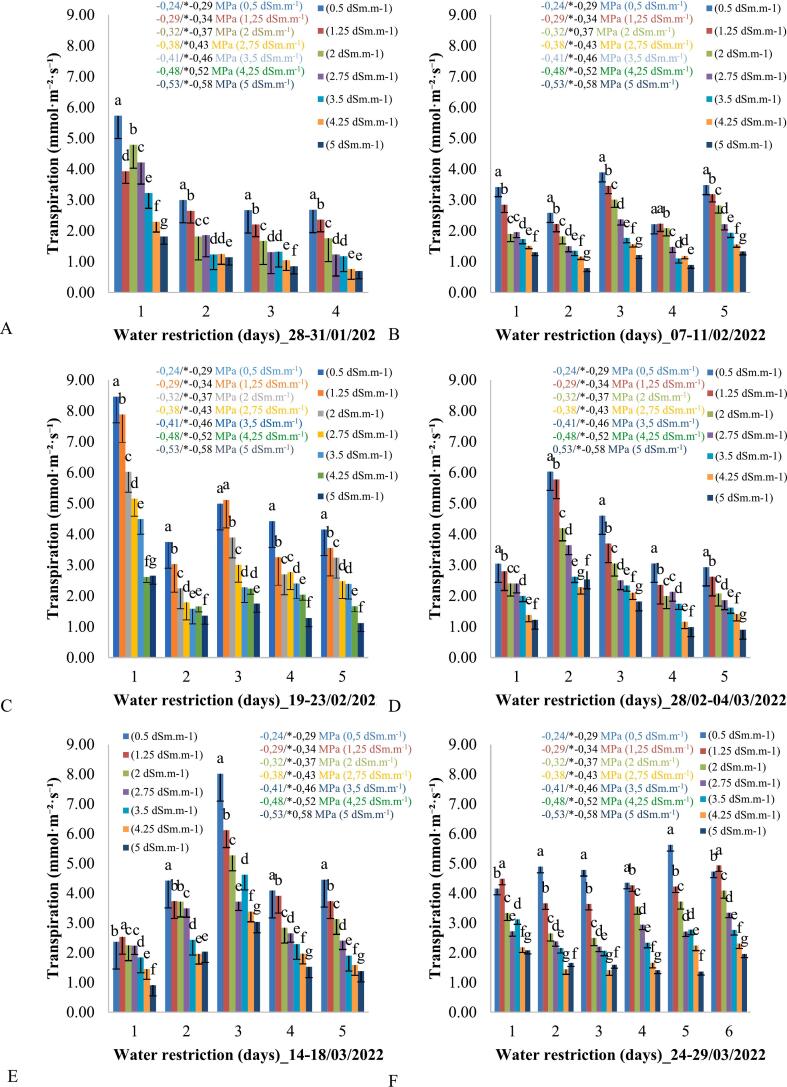
**A, B, C, D, E, F −** Leaf transpiration throughout the days of the week, from 6:00 am to 6:00 pm, in plants under different electrical conductivities and water potentials of irrigation water for the period from january 28 th to march 29 th from 2022. Equal letters between bars for the same day do not differ from each other by the Tukey test at 5 % probability (p < 0.05). * The water potential data were also obtained using the Scholander Pressure Chamber.

The graphs presented in Fig. 4 show a strong correlation between the water potentials measured by the portable chamber and the Scholander chamber, with determination coefficients (R^2^) above 0.95 for all levels of electrical conductivity (EC). This consistency in results validates the accuracy of the portable equipment and demonstrates its ability to provide reliable and replicable measurements even under different conditions and when using CO_2_.

Although inert gases have traditionally been used in pressure chamber systems (such as nitrogen or argon), the use of CO_2_ in the portable chamber offers practical and scientific advantages beyond its broad commercial availability. Carbon dioxide has a moderate density and suitable compressibility to generate controlled and stable pressures within the system. This stability is crucial to avoid fluctuations that could interfere with measurement accuracy. Furthermore, CO_2_ is easily liquefied at moderate pressures, enabling efficient storage in portable capsules, such as those used in the described equipment.

As the water potential becomes more negative, leaf transpiration tends to decrease significantly. This happens because, under lower water potential conditions, the force with which water is retained in the soil surpasses the plant's ability to extract it, leading to stomatal closure. This physiological response helps conserve water and prevent dehydration, but it also restricts the entry of CO_2_, thus limiting photosynthesis. Statistical analysis confirms that significant differences exist between the groups, as indicated by the different letters in the transpiration values for varying water potentials. Less negative potentials (−0.2 MPa) show higher transpiration, suggesting a condition of lower water stress. More negative potentials (−0.5 MPa) are associated with a drastic reduction in transpiration, indicating a critical state of water stress.

The Pressure Chamber equipment has proven to be an effective and accurate tool for measuring leaf water potential in plants under water stress. The equipment’s accuracy and precision are further confirmed by the close alignment of the measured values between the experimental units and its appropriate response to water stress during the period of restriction. Although the study was conducted with three experimental units (three plants) without replication, the data obtained were treated with statistical rigor appropriate to the experimental context. The small sample size was justified by the exploratory nature of the study, which aimed to validate the hardware's functionality under controlled conditions before its application in larger-scale studies with replication.

The statistical methodologies employed to validate the measurements obtained with the portable equipment were designed to ensure robust comparisons with the standard Scholander chamber. Initially, the data were analyzed using linear regression (Fig. 4), resulting in a high coefficient of determination (R^2^ = 0.9956), indicating a strong correlation between the water potentials measured by the two methods at different electrical conductivity (EC) levels of the irrigation water. This high correlation demonstrates the consistency and accuracy of the portable equipment.

**Fig. 6:**
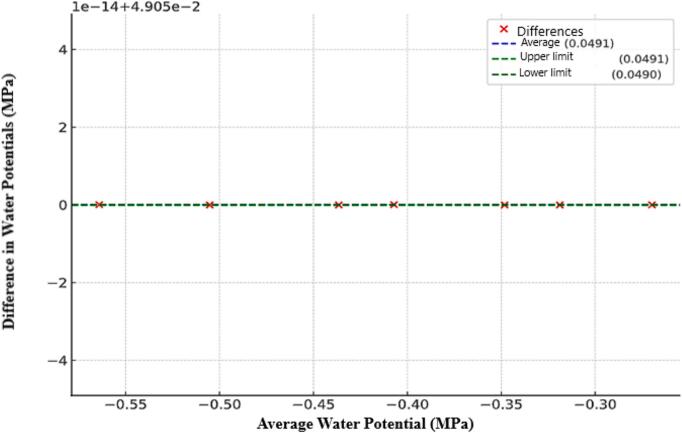
Bland-Altman plot comparing the water potential measurements obtained with the portable device and the Scholander chamber. The x-axis represents the mean of the measurements between the two methods, while the y-axis shows the difference between them. The dashed blue line indicates the mean difference between the methods, while the dashed green lines represent the limits of agreement at ± 1.96 standard deviations. The distribution of the points demonstrates the high agreement between the methods, with minimal systematic bias. (For interpretation of the references to colour in this figure legend, the reader is referred to the web version of this article.)

Additionally, the Bland-Altman analysis was used to assess the agreement and systematic bias between the methods (Fig. 6). The results indicated a mean bias of 0.0491 MPa, with extremely narrow limits of agreement, reflecting virtually no variability between the measurements of the two methods. These findings confirm the high precision of the portable equipment in replicating the measurements obtained with the Scholander chamber, even under varying conditions. The Bland-Altman statistical approach validates the reliability of the portable equipment and highlights its ability to provide accurate and consistent measurements in diverse experimental scenarios.

### 7.3 Characterization performance

The high precision of the hardware is demonstrated by its ability to provide reliable and consistent data on leaf water potential across different experimental settings, as evidenced by the strong linear correlation between measurements obtained by the equipment and the Scholander chamber (Fig. 4). This level of precision is further supported by the system's excellent repeatability, indicating minimal deviation between repeated measurements over different time periods (Fig. 5). Additionally, the hardware's rapid response time enhances its accuracy, with each measurement cycle from pressurization to image capture completed in approximately 4 s. These features collectively highlight the robustness of the hardware in delivering precise, repeatable, and efficient measurements, making it a reliable tool for advanced studies in plant ecophysiology.

### 7.4 Limitations hardware

The main limitation of the hardware lies in its restricted use to certain types of plants with leaf anatomy that includes the presence of a petiole. It is not suitable for very large leaves but is compatible with all plants whose anatomy is similar to that of eucalyptus leaves. Additionally, the maximum pressure the equipment can measure is 1 MPa.

### CRediT authorship contribution statement

**Willian Viana Campos:** Conceptualization, Data curation, Formal analysis, Funding acquisition, Investigation, Methodology, Project administration, Resources, Software, Supervision, Validation, Visualization, Writing – original draft, Writing – review & editing. **Jose Teixeira Filho:** Visualization, Validation, Supervision, Software, Resources, Project administration, Methodology, Investigation, Funding acquisition, Formal analysis, Data curation, Conceptualization. **Alcebiades Rebouças São José:** Visualization, Validation, Supervision, Software, Resources, Project administration, Methodology, Investigation, Funding acquisition, Formal analysis, Data curation, Conceptualization.

## Declaration of competing interest

The authors declare that they have no known competing financial interests or personal relationships that could have appeared to influence the work reported in this paper.
